# AI-ECG Risk Stratification for Atrial Fibrillation: Real-World Performance and Explainability

**DOI:** 10.1016/j.jacadv.2026.103036

**Published:** 2026-07-22

**Authors:** Kouki Matsuo, Yoshihiro Sobue, Taiji Miyake, Kazuya Takeda, Eiichi Watanabe, Hitoshi Matsuo, Hideo Izawa

**Affiliations:** a Department of Cardiology, Fujita Health University Bantane Hospital, Nagoya, Japan; b Department of Cardiovascular Medicine, Gifu Heart Center, Gifu, Japan; c Graduate School of Health Sciences, Fujita Health University Graduate School, Toyoake, Japan; d Department of Cardiology, Fujita Health University, Toyoake, Japan

**Keywords:** atrial fibrillation, artificial intelligence, electrocardiography, external validation, machine learning, risk stratification

## Abstract

**Background:**

Artificial intelligence–enabled electrocardiography (AI-ECG) has emerged as a potential method for identifying atrial fibrillation (AF) from sinus rhythm. However, its clinical utility and interpretability in routine practice remain uncertain.

**Objectives:**

The objective of the study was to assess the performance and explainability of an AI-integrated ECG system for AF risk stratification in a multicenter real-world cohort.

**Methods:**

We enrolled 665 patients aged ≥40 years who underwent 12-lead ECGs using an AI-enabled electrocardiograph (FCP-9900). The device automatically assigned AF risk into 4 categories (low, mid-low, mid-high, and high). Machine-learning models—support vector machine, adaptive boosting, and artificial neural networks—were developed using clinical and ECG-derived variables. Internal validation used stratified 10-fold cross-validation, and external validation used an independent cohort. Feature contributions were assessed with SHapley Additive exPlanations.

**Results:**

AF prevalence increased across AI-ECG risk categories, with significantly higher odds in the mid-high and high groups vs low. Model 2, which incorporated CHADS_2_ and CHA_2_DS_2_-VASc scores, achieved strong discrimination in internal and external validation (support vector machine AUC 1.00; adaptive boosting 0.97-0.98; artificial neural network 0.89-0.95), outperforming AI-ECG alone (area under the receiver operating characteristic curve: 0.64-0.69). SHapley Additive exPlanations analysis showed CHA_2_DS_2_-VASc as the most influential predictor, whereas AI-ECG provided modest incremental value.

**Conclusions:**

AI-ECG provides rapid, low-cost AF risk estimation from a single sinus rhythm ECG; however, its predictive performance is modest compared with clinical scores. At present, AI-ECG may complement, but not replace, traditional risk stratification.

Atrial fibrillation (AF) is the most common sustained cardiac arrhythmia and a major contributor to stroke, heart failure, and mortality. Because AF is frequently asymptomatic and paroxysmal, a substantial proportion of cases remain undiagnosed in routine clinical practice. Identifying individuals with latent or previously unrecognized AF therefore represents an important clinical challenge, particularly in settings where prolonged rhythm monitoring is not readily available.^1^, ^2^, ^3^

Artificial intelligence (AI)–enabled electrocardiography (ECG) (AI-ECG) has emerged as a promising approach for detecting latent cardiovascular conditions from standard 12-lead ECGs obtained during sinus rhythm (SR). Prior studies have demonstrated that deep learning models can identify individuals with AF or structural heart disease despite the absence of overt rhythm abnormalities on the surface ECG.^4^^,^^5^ However, much of the existing evidence is derived from algorithm development, technical validation, or controlled research settings, limiting insight into how such systems perform when implemented in routine clinical practice.

Indeed, although AI-ECG systems for AF risk assessment have been developed and their technical feasibility has been demonstrated, their performance has not been sufficiently validated in real-world clinical environments. In particular, evidence regarding the clinical performance of commercially available AI-ECG systems when integrated into everyday workflows remains limited. Moreover, enthusiasm for AI-based AF detection must be tempered by practical considerations. Established clinical risk scores, such as CHA_2_DS_2_-VASc, remain widely available, inexpensive, and strongly associated with AF prevalence in routine care. Whether AI-ECG provides clinically meaningful incremental information beyond readily accessible clinical variables therefore remains uncertain. In addition, many prior investigations have emphasized algorithmic performance metrics without addressing explainability, which is increasingly recognized as essential for clinical adoption and trust.^6^

Therefore, we conducted a multicenter cross-sectional study to evaluate the real-world performance and explainability of a commercially deployed AI-ECG system for AF risk stratification using SR ECGs obtained during routine clinical care. Importantly, this investigation was designed to focus on associations with prevalent AF rather than predicting incident AF or future clinical outcomes. We assessed the incremental value of AI-ECG relative to established clinical risk scores and explored model behavior using SHapley Additive exPlanations (SHAP) to clarify the relative contributions of clinical and ECG-derived features.

## Methods

### Study design and population

This multicenter, cross-sectional study was conducted at 2 cardiovascular institutions in Japan: Fujita Health University Bantane Hospital and Gifu Heart Center. The study enrolled patients aged ≥40 years who were undergoing evaluation or follow-up for cerebrovascular and cardiovascular diseases as outpatients. All participants underwent a standard 10-second, 12-lead ECG using the FCP-9900 device (Fukuda Denshi) during the study period between January 2024 and March 2025. AF was defined as an irregular rhythm without identifiable P waves lasting ≥30 seconds and considered present when documented on a standard 12-lead ECG.

### Inclusion and exclusion criteria

Patients were eligible for inclusion if they were aged ≥40 years and had SR on ECG. The exclusion criteria mirrored those used in the development and validation of the original model,^5^ and included: 1) paced rhythms; 2) ECGs obtained after catheter ablation or cardiac surgery; 3) mitral stenosis or prosthetic heart valves; 4) cardiogenic cerebral embolism in the non-AF group; 5) arrhythmias listed in the predefined exclusion list; and 6) poor-quality ECGs due to lead misplacement or noise. The study protocol was approved by the institutional review boards of both participating institutions (HM24-226). Written informed consent was obtained from all participants, and the study was conducted in accordance with the principles outlined in the Declaration of Helsinki.

### Device description and AI-based risk stratification

The FCP-9900 is a commercially available electrocardiograph integrated with an edge-deployable AI algorithm designed to predict AF risk from SR ECGs in real time. The AI model incorporated into this device employs a lightweight convolutional neural network optimized for resource-constrained environments. Its performance and technical specifications have been previously validated in an independent study.^5^ In brief, the algorithm processes 1-second averaged waveforms from 12-lead ECGs, which are converted into binary images after signal preprocessing. These images are arranged in a composite matrix and input into the convolutional neural network to identify subtle morphological patterns indicative of atrial electrical remodeling. Based on the output, the model stratifies AF risk into 4 categories: low, mid-low, mid-high, and high. The prediction is completed in approximately 2 seconds on the device.

### Machine learning analysis

Supervised machine learning models were developed to identify predictors of AF using demographic, clinical, and ECG-derived variables. Three model configurations were evaluated: model 1, consisting of 31 baseline features; model 2, which added CHADS_2_ and CHA_2_DS_2_-VASc scores for a total of 33 features; and model 3, comprising the same 33 features as model 2 but excluding the AI-ECG output (32 features) (Supplemental Figure 1).

Continuous variables were normalized, and categorical variables were one-hot encoded before model training. Three supervised classifiers were implemented: a linear-kernel support vector machine (SVM), adaptive boosting (AdaBoost) using decision-tree base learners, and a fully connected artificial neural network (ANN) were performed using MATLAB R2024b. A linear-kernel SVM was selected to improve interpretability and reduce the risk of overfitting given the modest sample size, whereas the ANN architecture was used to explore potential nonlinear relationships among clinical and ECG-derived variables. Internal validation was performed within the derivation cohort from Gifu Heart Center using stratified 10-fold cross-validation to assess model stability and reproducibility. For external validation, the final models were retrained on the full derivation data set and evaluated in an independent cohort from Fujita Health University Bantane Hospital to examine generalizability across institutions. Model performance was assessed by calculating the area under the receiver operating characteristic curve (AUC), sensitivity, specificity, and overall accuracy in both cohorts. To enhance interpretability, SHAP values were computed to quantify the relative contribution of each feature, with global importance visualized using summary plots and individualized risk attribution illustrated with force plots. To assess multicollinearity among predictor variables, variance inflation factor (VIF) analysis was performed for the 33-feature model.

### Statistical analysis

Continuous variables are presented as mean ± SD, and categorical variables as absolute numbers and percentages. Between-group comparisons were conducted using the Student’s t-test or one-way analysis of variance for continuous variables, and the chi-square test or Fisher exact test for categorical variables, as appropriate. When expected cell counts were <5, Fisher exact test was applied. All statistical tests were 2-tailed, and a *P* value < 0.05 was considered statistically significant. Analyses were performed using JMP Pro (version 17; SAS Institute) and R software (version 4.2.1; R Foundation for Statistical Computing). The discriminatory performance of the models in the external validation cohort was evaluated using the AUC. To assess statistical uncertainty, 95% CIs for the AUCs were calculated using a bootstrapping method with 2,000 iterations by random sampling with replacement from the external validation data set. As an exploratory analysis, net reclassification improvement was calculated to assess the incremental value of AI-ECG beyond conventional clinical risk scores, based on predefined risk categories and AF status.

## Results

### Patient characteristics

From January 2024 to March 2025, a total of 836 outpatients with stable cardiovascular or cerebrovascular disease and no symptoms were screened at Fujita Health University Bantane Hospital and Gifu Heart Center (Supplemental Figure 2). Of these, 171 patients (20.4%) were excluded for the following predefined reasons: age <40 years (n = 15), ongoing treatment with antiarrhythmic drugs (n = 36), poor ECG quality or frequent extrasystoles (n = 26), paced rhythm (n = 39), prior catheter ablation (n = 45), and prior cardiac surgery (n = 10). The remaining 665 patients were included in the final analysis. The patient characteristics are summarized in Table 1. The mean age was 68.5 ± 12.2 years, and 285 patients (42.8%) were women. Hypertension was present in 374 patients (56.2%), diabetes mellitus in 145 (21.8%), and congestive heart failure in 74 (11.2%). The mean estimated glomerular filtration rate was 65.7 ± 19.6 mL/min/1.73 m^2^, and the median N-terminal pro–B-type natriuretic peptide level was 114 pg/mL (IQR: 51-291 pg/mL). On baseline ECG, the mean PR interval was 173 ± 30 ms, QRS duration 103 ± 17 ms, QT interval 401 ± 34 ms, and corrected QT interval (QTc) 423 ± 26 ms. Echocardiographic measurements showed a mean left ventricular ejection fraction of 60.0% ± 10.8%, left atrial diameter of 36.5 ± 6.3 mm, and left atrial volume index of 32.0 ± 12.2 mL/m^2^.

**Table 1:** Patient Characteristics (N = 665)

Age (years)	68.5 ± 12.2
≥65 y	436 (65.5)
≥75 y	250 (37.6)
Female, n (%)	285 (42.8)
BMI	23.5 ± 3.7
Comorbidities, n (%)	
Congestive heart failure	74 (11.2)
Hypertension	374 (56.2)
Diabetes mellitus	145 (21.8)
Stroke or TIA	9 (1.4)
Vascular disease	236 (35.9)
CHADS_2_	1.4 ± 1.1
CHA_2_DS_2_-VASc	2.4 ± 1.4
Systolic blood pressure (mmHg)	133.6 ± 20.2
Diastolic blood pressure (mmHg)	74.7 ± 13.0
Heart rate (beat/minutes)	68.2 ± 11.5
Laboratory data	
RBC–10^4^/μL	438 ± 61
Hemoglobin–g/dL	13.4 ± 1.9
BUN–mg/dL	17.3 ± 5.2
Serum creatinine–mg/dL	0.94 ± 0.79
eGFR–mL/min/1.73 m^2^	65.7 ± 19.6
NT-pro BNP–pg/mL	114 (51-291)
Electrocardiogram	
RR (ms)	905 ± 150
PR (ms)	173 ± 30
QRS (ms)	103 ± 17
QT (ms)	401 ± 34
QTc	423 ± 26
Echocardiography	
Ejection fraction (%)	60.0 ± 10.8
Left atrial diameter (mm)	36.5 ± 6.3
LAVi (mL/m^2^)	32.0 ± 12.2
IVS (mm)	9.5 ± 1.9
PW (mm)	9.2 ± 1.5

Values are mean ± SD, n (%), or median (25th and 75th percentile).

BMI = body mass index; BUN = blood urea nitrogen; eGFR = estimated glomerular filtration rate; IVS = interventricular septum; LAVi = left atrial volume index; NT-proBNP = N-terminal pro–B-type natriuretic peptide; PW = posterior wall; RBC = red blood cell; TIA = transient ischemic attack.

**Figure 1:**
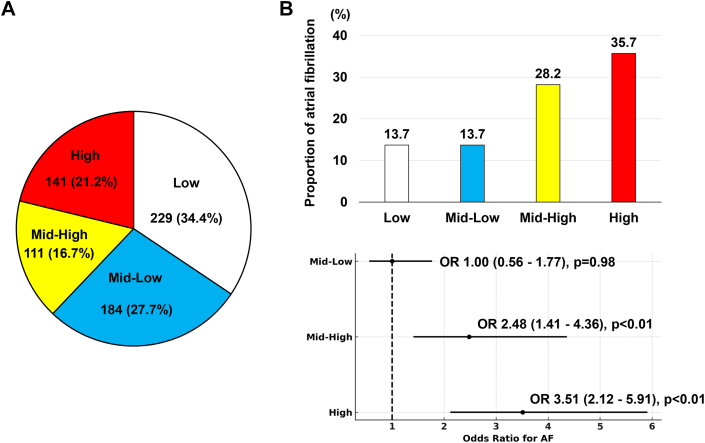
**Distribution of Artificial Intelligence–Enabled Electrocardiography Risk Categories and Association With Atrial Fibrillation** (A) Distribution of AI-ECG risk categories. (B) ORs for prevalent AF across AI-ECG risk categories, using the Low-risk group as the reference category. AF = atrial fibrillation.

Based on the AI-ECG risk classification, 229 patients (34.4%) were categorized as low risk, 184 (27.7%) as mid-low risk, 111 (16.7%) as mid-high risk, and 141 (21.2%) as high risk (Figure 1A). Across the 4 AI-ECG risk categories (low, mid-low, mid-high, and high), patient profiles demonstrated significant progressive gradients (Supplemental Table 1). Age increased from 66 ± 13 years in the low group to 71 ± 11 years in the high group (*P* < 0.01). Congestive heart failure prevalence rose from 6% to 13% (*P* < 0.01). CHADS_2_ and CHA_2_DS_2_-VASc scores increased from 1.2 ± 1.1 to 1.6 ± 1.2 and from 2.1 ± 1.3 to 2.7 ± 1.5, respectively (*P* < 0.01 for both). Renal function declined, with estimated glomerular filtration rate decreasing from 69 ± 20 to 61 ± 21 mL/min/1.73 m^2^ (*P* < 0.01), whereas N-terminal pro–B-type natriuretic peptide levels increased from 93 (45–179) pg/mL to 152 (63–428) pg/mL (*P* < 0.01). On ECG, PR interval prolonged from 167 ± 28 to 184 ± 32 ms (*P* < 0.01) and QT interval lengthened from 389 ± 28 to 416 ± 35 ms (*P* < 0.01), whereas QTc remained similar across groups (*P* = 0.07). Heart rate decreased from 71 ± 10 to 64 ± 12 bpm (*P* < 0.01), and diastolic blood pressure was modestly lower in the high vs low group (72 ± 12 vs 76 ± 14 mmHg; *P* = 0.02). Echocardiography showed larger left atrial dimensions with increasing AI-ECG risk (left atrial diameter: 35 ± 5-38 ± 7 mm; left atrial volume index: 29 ± 10-37 ± 12 mL/m^2^; *P* < 0.01 for both). Left ventricular ejection fraction differed modestly across groups (*P* = 0.01).

Among the 665 patients, 137 (20.6%) had a prior diagnosis of AF. AF prevalence increased markedly with higher AI-ECG risk categories, with unadjusted ORs vs low of 1.00 (mid-low; 95% CI: 0.56-1.77; *P* = 0.98), 2.48 (mid-high; 95% CI: 1.41-4.36; *P* < 0.01), and 3.51 (high; 95% CI: 2.12-5.91; *P* < 0.01) (Figure 1B).

### Model performance

**Figure 2:**
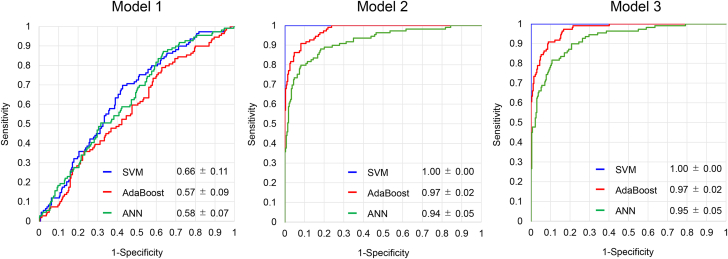
**Area Under the Curve Performance for Internal Validation** Internal validation results for support vector machine (SVM), adaptive boosting (AdaBoost), and artificial neural network (ANN) across model 1, model 2, and model 3.

In the internal validation cohort, classifier performance varied across model configurations (Table 2). For the SVM, model 1 showed limited discriminative ability (accuracy 71.2%; AUC: 0.66), whereas both model 2 and model 3 achieved perfect classification, with all performance metrics including accuracy, precision, recall, specificity, and AUC reaching 1.00 (Figure 2). For AdaBoost, accuracy improved from 60.9% (AUC: 0.57) in model 1 to 93.6% (AUC: 0.97) in model , and 93.9% (AUC: 0.97) in model 3. Similarly, the ANN improved from 66.1% (AUC: 0.58) in model 1 to 90.2% (AUC: 0.94) in model 2, and 93.1% (AUC: 0.95) in model 3. In comparison, the AI-ECG score alone demonstrated an accuracy of 64.6% (AUC: 0.64), the CHA_2_DS_2_-VASc score alone 64.3% (AUC: 0.71), and the CHADS_2_ score alone 56.1% (AUC: 0.54).

**Table 2:** Performance Metrics for Internal Validation

	Accuracy	Precision	Recall	NPV	Specificity	F-Score	AUC
SVM							
Model 1	71.2 ± 2.4	20.0 ± 40.0	1.9 ± 3.8	71.4 ± 1.5	99.3 ± 2.2	3.5 ± 7.0	0.66 ± 0.11
Model 2	100 ± 0	100 ± 0	100 ± 0	100 ± 0	100 ± 0	100 ± 0	1.0 ± 0
Model 3	100 ± 0	100 ± 0	100 ± 0	100 ± 0	100 ± 0	100 ± 0	1.0 ± 0
AdaBoost							
Model 1	60.9 ± 7.3	33.6 ± 13.8	38.6 ± 18.8	74.0 ± 2.8	69.9 ± 15.8	34.2 ± 12.9	0.57 ± 0.09
Model 2	93.9 ± 4.4	87.1 ± 11.1	95.4 ± 4.6	98.1 ± 1.9	93.3 ± 6.6	90.5 ± 6.1	0.97 ± 0.02
Model 3	93.6 ± 4.6	86.1 ± 10.2	94.5 ± 4.5	97.6 ± 1.9	93.3 ± 5.4	89.8 ± 6.9	0.97 ± 0.02
ANN							
Model 1	66.1 ± 5.7	38.6 ± 19.1	25.6 ± 14.5	73.4 ± 3.3	82.5 ± 9.5	28.8 ± 13.3	0.58 ± 0.07
Model 2	90.2 ± 5.7	81.0 ± 11.8	88.9 ± 8.1	95.4 ± 3.4	90.7 ± 6.9	84.3 ± 8.3	0.94 ± 0.05
Model 3	93.1 ± 4.4	87.1 ± 10.5	90.8 ± 5.8	96.2 ± 2.5	94.1 ± 5.0	88.7 ± 6.9	0.95 ± 0.05
AI-ECG	64.6 ± 6.3	40.2 ± 11.8	56.0 ± 19.7	79.9 ± 6.5	68.1 ± 6.9	46.4 ± 14.8	0.64 ± 0.09
CHA_2_DS_2_-VASc	64.3 ± 7.7	42.3 ± 9.0	58.8 ± 10.6	79.8 ± 5.2	66.5 ± 8.8	49.0 ± 9.1	0.71 ± 0.07
CHADS_2_	56.1 ± 6.7	31.2 ± 7.4	42.4 ± 10.9	72.4 ± 4.6	61.7 ± 8.4	35.7 ± 8.3	0.54 ± 0.06

AdaBoost = adaptive boosting; AI-ECG = artificial intelligence–enabled electrocardiography; ANN = artificial neural network; AUC = area under the receiver operating characteristic curve; NPV = negative predictive value; SVM = support vector machine.

**Figure 3:**
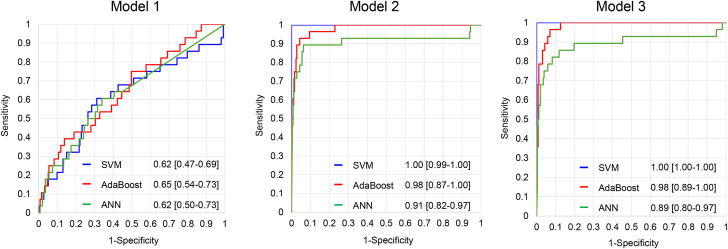
**Area Under the Curve Performance for External Validation** External validation results for support vector machine (SVM), adaptive boosting (AdaBoost), and artificial neural network (ANN) across model 1, model 2, and model 3.

In the external validation cohort, performance was consistently high for models 2 and 3 (Table 3). The SVM achieved an accuracy of 99.6% (AUC: 1.00) in model 2 and 98.9% (AUC: 1.00) in model 3, compared with 90.0% (AUC: 0.62) in model 1. AdaBoost improved from 78.6% (AUC: 0.65) in model 1 to 88.3% (AUC: 0.98) in model 2 and 87.9% (AUC: 0.98) in model 3 (Figure 3). The ANN increased from 73.2% (AUC: 0.62) in model 1 to 87.2% (AUC: 0.91) in model 2 and 87.9% (AUC: 0.89) in model 3. By comparison, AI-ECG alone achieved an accuracy of 80.1% (AUC: 0.69), CHA_2_DS_2_-VASc alone 80.8% (AUC: 0.80), and CHADS_2_ alone 79.4% (AUC: 0.67).

**Table 3:** Performance Metrics for External Validation

	Accuracy	Precision	Recall	NPV	Specificity	F-Score	AUC
SVM							
Model 1	90.0	50.0	3.6	90.3	99.6	6.7	0.62
Model 2	99.6	100	96.4	99.6	100	98.2	1.00
Model 3	98.9	100	89.3	98.8	100	94.3	1.00
AdaBoost							
Model 1	78.6	20.0	40.7	92.9	82.6	26.8	0.65
Model 2	87.9	45.2	100	100	86.6	62.2	0.98
Model 3	88.3	45.8	96.4	99.5	87.4	62.1	0.98
ANN							
Model 1	73.2	15.7	40.7	92.4	76.7	22.7	0.62
Model 2	87.2	43.1	89.3	98.7	87.0	58.1	0.91
Model 3	87.9	44.4	85.7	98.2	88.1	58.5	0.89
AI-ECG	80.1	23.1	42.9	93.0	84.2	30.0	0.69
CHA_2_DS_2_-VASc	80.8	29.7	67.9	95.9	82.2	41.3	0.80
CHADS_2_	79.4	20.0	35.7	92.2	84.2	25.6	0.67

Abbreviations as in Table 2.

There was no material difference in discriminative performance between model 2 and model 3 across internal and external validation. To further evaluate feature redundancy, VIF analysis was performed for the 33-feature model (Supplemental Table 2). Substantial multicollinearity was observed, particularly for the CHADS_2_ score (VIF = 15.5) and CHA_2_DS_2_-VASc score (VIF = 9.9), supporting the presence of feature redundancy among the predictor variables.

**Figure 4:**
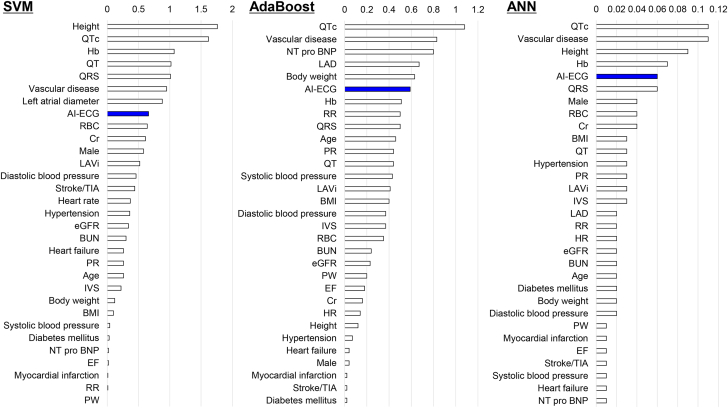
**SHapley Additive exPlanations Summary Plot for Model 1** Mean absolute SHapley Additive exPlanations (SHAP) values are shown for all predictors in Model 1 across the 3 classifiers (SVM, AdaBoost, and ANN). AdaBoost = adaptive boosting; ANN = artificial neural network; BMI = body mass index; BUN = blood urea nitrogen; Cr = serum creatinine; EF = ejection fraction; eGFR = estimated glomerular filtration rate; Hb = hemoglobin; HR = heart rate; IVS = interventricular septum; LAD = left anterior descending coronary artery; LAVi= left atrial volume index; NT-proBNP = N-terminal pro–B-type natriuretic peptide; PW = posterior wall; RBC = red blood cell; SVM = support vector machine; TIA = transient ischemic attack.

Explainability analyses using SHAP showed distinct patterns between model 1 and model 2. In model 1 (without CHADS_2_ or CHA_2_DS_2_-VASc scores), no single dominant predictor emerged across classifiers. Instead, AF discrimination relied on a constellation of modest contributors, with QTc and QRS duration exhibiting relatively greater importance (SVM: 1.62 and 1.01; AdaBoost: 1.08 and 0.50; ANN: 0.11 and 0.06). The AI-ECG score also contributed modestly (0.66, 0.59, and 0.06, respectively) (Figure 4, Supplemental Table 3).

**Figure 5:**
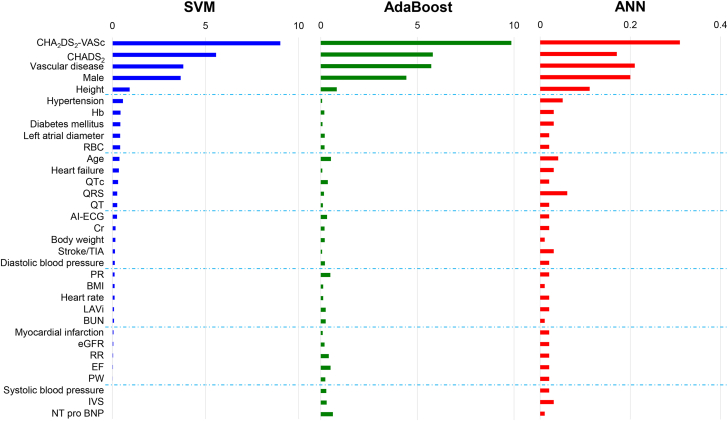
**SHAP Summary Plot for Model 2** Mean absolute SHapley Additive exPlanations (SHAP) values are shown for all predictors in model 2 across the three classifiers (SVM, AdaBoost, and ANN). Abbreviations as in Figure 4.

By contrast, in model 2—where clinical risk scores were incorporated—CHA_2_DS_2_-VASc became overwhelmingly the most influential feature across all classifiers, with the mean absolute SHAP values of 9.02 (SVM), 9.86 (AdaBoost), and 0.31 (ANN). In this setting, the AI-ECG score contributed less prominently, with SHAP values of 0.25, 0.33, and 0.02, respectively (Figure 5, Supplemental Table 4). These differences in feature importance were reflected in model performance. In internal validation, model 1 achieved modest discrimination (SVM: AUC 0.66; AdaBoost: 0.57; ANN: 0.58), whereas model 2 consistently demonstrated excellent performance across classifiers (SVM: 1.00; AdaBoost: 0.97; ANN: 0.95). Similar patterns were observed in external validation, where AUCs remained high for model 2 (SVM: 1.00; AdaBoost: 0.98; ANN: 0.89) but were lower in model 1 (SVM: 0.62; AdaBoost: 0.65; ANN: 0.62) (Supplemental Figure 3). Net reclassification improvement analysis demonstrated minimal improvement in risk classification (net reclassification improvement = 0.007; *P* = 0.92), indicating limited incremental value of AI-ECG beyond conventional clinical risk scores (Supplemental Table 5).

**Central Illustration:**
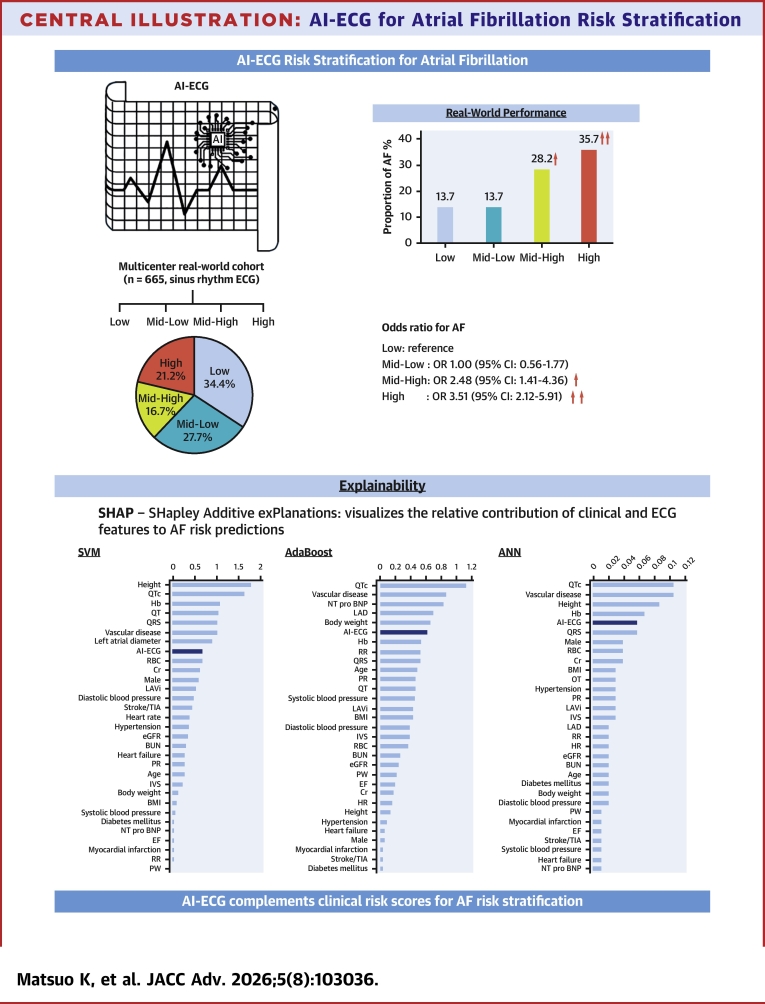
**AI-ECG for Atrial Fibrillation Risk Stratification** AI-ECG enables risk stratification for atrial fibrillation in a multicenter real-world cohort. Higher AI-ECG risk categories are associated with an increased prevalence of AF, with progressively higher odds ratios compared with the low-risk group. Receiver operating characteristic analysis demonstrates the performance of AI-ECG relative to conventional clinical risk scores. SHAP (SHapley Additive exPlanations) analysis illustrates the contribution of both clinical variables and ECG features to model predictions, supporting the interpretability of AI-ECG. These findings suggest that AI-ECG provides a simple and explainable tool that complements established clinical risk scores for AF risk assessment. AdaBoost = adaptive boosting; AF = atrial fibrillation; AI-ECG = artificial intelligence–enabled electrocardiography; ANN = artificial neural network; BMI = body mass index; BUN = blood urea nitrogen; Cr = serum creatinine; EF = ejection fraction; eGFR = estimated glomerular filtration rate; Hb = hemoglobin; HR = heart rate; IVS = interventricular septum; LAD = left anterior descending coronary artery; LAVi= left atrial volume index; NT-proBNP = N-terminal pro–B-type natriuretic peptide; PW = posterior wall; RBC = red blood cell; SVM = support vector machine; TIA = transient ischemic attack.

## Discussion

### Clinical implications of an AI-ECG for AF risk detection

This multicenter, cross-sectional study is the first to validate an AI-ECG system for AF risk stratification during routine cardiovascular evaluation in real-world practice. The AI algorithm, which analyzes SR ECGs, stratified patients into 4 risk categories, and the prevalence of clinically diagnosed AF increased stepwise across these groups. Notably, only the mid-high category remained independently associated with AF after adjustment for conventional risk factors, suggesting that the AI algorithm may capture electrophysiological signatures associated with AF beyond established clinical markers.^3^

Subclinical atrial fibrillation has been increasingly recognized as clinically meaningful, as even brief device-detected atrial high-rate episodes are associated with higher risks of ischemic stroke and subsequent clinical AF.^1^^,^^2^ In addition, large ambulatory ECG monitoring studies have shown that previously unrecognized AF is not uncommon in older adults.^7^^,^^8^ In the Atherosclerosis Risk in Communities study using patch-based monitoring, newly detected AF was observed in approximately 2% to 3% of asymptomatic elderly individuals, underscoring the limitations of intermittent ECG screening. Similarly, the KP-RHYTHM cohort demonstrated that prolonged wearable monitoring can reveal a substantial burden of previously undetected AF. Collectively, these observations emphasize the clinical importance of undetected or subclinical AF and highlight the need for simple, low-burden tools that can enrich the identification of at-risk individuals.

Against this background, our findings suggest that AI-based ECG risk stratification may serve as a practical adjunct for identifying patients with unrecognized AF susceptibility.^6^ In settings where extended monitoring is impractical, the ability to derive risk information from a single 10-second ECG may help guide decisions regarding further rhythm evaluation. The observation that the mid-high—but not the high—risk group retained significance after multivariable adjustment likely reflects the higher burden of comorbidities within the high-risk category, which may attenuate its independent association. This pattern warrants confirmation in larger longitudinal cohorts and highlights the need to refine how AI-derived risk categories should be interpreted in diverse clinical populations.

### Comparative predictive value of AI-ECG and CHA_2_DS_2_-VASc

In the present study, machine learning classifiers—including SVMs, AdaBoost, and ANNs—achieved high accuracy in identifying AF when both clinical and ECG-derived features were incorporated. Performance remained consistently strong across internal and external validation, demonstrating the feasibility of integrating advanced algorithms for AF risk assessment in real-world cohorts.

When the AI-ECG output was examined in isolation, however, its discriminatory ability was more modest. SHAP-based explainability confirmed that its predictive contribution was consistently lower than that of established clinical variables, with CHA_2_DS_2_-VASc emerging as the dominant feature across all models. These findings indicate that although AI-ECG may provide useful electrophysiologic information from a single SR tracing, it cannot replace conventional clinical risk stratification.

This pattern aligns with prior large-scale cohort studies showing that CHADS_2_ and CHA_2_DS_2_-VASc scores reliably predict incident AF and thromboembolic events,^9^ and that higher scores are associated with subclinical AF in device-based studies.^1^^,^^2^ Recent randomized trials of anticoagulation for device-detected subclinical AF further underscore that thromboembolic risk—and the potential benefit of intervention—is primarily driven by underlying clinical risk factors.^10^^,^^11^ These data collectively support the central role of CHA_2_DS_2_-VASc in AF-related risk assessment and help explain why it remained the strongest predictor in our models, whereas the AI-ECG signal provided only modest incremental value.

Against this background, our study offers a complementary perspective: although AI-ECG alone is insufficient for therapeutic decision-making and demonstrated limited incremental value beyond conventional clinical risk scores, it represents a practical, low-burden tool that may help identify individuals who could benefit from further rhythm surveillance (Central Illustration). Unlike conventional clinical risk scores, which require integration of multiple patient-specific factors, AI-ECG provides an immediate output derived from a single 10-second ECG and may be particularly useful in primary care or community settings where detailed clinical data are not readily available. Future studies incorporating multimodal data, including wearable rhythm monitoring technologies, may further enhance the identification of individuals at risk for AF and improve risk stratification strategies.

### Study limitations

Several limitations of this study should be acknowledged. First, this was a cross-sectional analysis and therefore cannot establish causal relationships between the AI-derived risk classification and the presence of AF. Because of the cross-sectional design and the use of clinically recognized AF as the outcome, the models should be interpreted as identifying correlates of existing AF rather than predicting future AF risk. Longitudinal studies are needed to assess the predictive value of the algorithm for incident AF and clinical outcomes such as stroke. Second, although the AI-ECG algorithm was applied in a real-world setting, the study population was limited to patients undergoing cardiovascular evaluation or follow-up at 2 specialized centers in Japan. As such, generalizability to broader populations, including asymptomatic individuals or those in primary care, may be limited. Third, the diagnosis of AF was based on existing clinical records, without uniform long-term rhythm monitoring across all participants. Therefore, some cases of paroxysmal or brief subclinical AF may have gone undetected, potentially underestimating the true association between AI risk categories and AF prevalence. Fourth, although we included demographic variables in the SHAP analysis, unmeasured confounding factors—such as left atrial size, natriuretic peptide levels, or burden of supraventricular ectopy—could have influenced the results. Future studies integrating clinical, imaging, and biomarker data may enhance risk stratification performance. Fifth, the SVM classifier in model 2 achieved an AUC of 1.00 in both internal and external validation. Although this likely reflects strong separation between AF and non-AF phenotypes in our high-risk, referral-center population, such perfect performance is unusual and should be interpreted with caution. This may be partly explained by feature redundancy and multicollinearity, as composite clinical risk scores (CHA_2_DS_2_-VASc and CHADS_2_) were included alongside their component variables. VIF analysis demonstrated substantial multicollinearity, particularly for the CHADS_2_ score (VIF = 15.5) and CHA_2_DS_2_-VASc score (VIF = 9.9). Consistent with this interpretation, exclusion of these composite scores resulted in a marked reduction in model performance. The marked reduction in performance after exclusion of these composite scores supports this interpretation. In addition, spectrum bias and site effects due to similar inclusion criteria and case mix across the 2 centers may have contributed to the observed results. No site-specific harmonization or calibration procedures were applied before external validation, and the models were directly evaluated using data from the independent external cohort to assess generalizability under real-world conditions. Because the present study was designed as a cross-sectional association analysis rather than a prognostic prediction model, calibration analyses such as calibration slope or Brier score were not performed. Therefore, some degree of overfitting cannot be entirely excluded, and these findings should be considered hypothesis-generating until validated in larger, more diverse cohorts. Finally, the performance of the FCP-9900 AI algorithm has not been externally validated in other geographic or ethnic populations. Additional multicenter and international validation studies are warranted to confirm the reproducibility and scalability of the current findings.

## Conclusions

In this multicenter real-world study, an AI-integrated ECG system enabled efficient risk stratification for AF using a single SR ECG. Although higher AI-ECG risk categories were associated with prevalent AF, its predictive contribution was modest when compared with traditional clinical factors. Explainability analysis demonstrated that the CHA_2_DS_2_-VASc score remained the dominant determinant of AF risk, with the AI-ECG score providing incremental but limited value. These findings suggest that AI-ECG may serve as a practical adjunct to identify patients who merit further rhythm evaluation, whereas established clinical risk factors continue to drive AF prediction. Prospective studies are needed to define its prognostic role in incident AF.

Perspectives **Competency in Medical Knowledge**

• AI-ECG allows rapid, low-burden estimation of AF risk from a single SR ECG in routine clinical practice.
• In real-world cohorts, AI-ECG risk categories are associated with prevalent AF; however, established clinical risk scores such as CHA_2_DS_2_-VASc remain the dominant predictors of AF risk.
• AI-ECG should therefore be considered a complementary tool to enrich AF risk stratification and guide decisions regarding further rhythm monitoring, rather than a replacement for conventional clinical assessment.

**Translational Outlook**

• Prospective longitudinal studies are needed to determine whether AI-ECG risk stratification can predict incident AF and AF-related outcomes, including stroke, beyond established clinical risk scores.
• Integration of AI-ECG with multimodal data—such as echocardiography, biomarkers, and wearable rhythm monitoring—may enhance predictive performance and clinical utility.
• Future research should focus on defining optimal clinical pathways in which AI-ECG–guided risk assessment can be implemented efficiently and safely in primary care and population-based screening settings.

## Funding support and author disclosures

This research did not receive any specific grant from funding agencies in the public, commercial, or not-for-profit sectors. Dr Hitoshi Matsuo reports Speakers Bureau of Abbott Vascular, Philips, HeartFlow, Amgen, and Zeon Medical. All other authors have reported that they have no relationships relevant to the contents of this paper to disclose.
