# Supplementary material for: AI-ECG Risk Stratification for Atrial Fibrillation: Real-World Performance and Explainability
Source: JACC Adv. 2026 Jul 22;5(8):103036. doi: 10.1016/j.jacadv.2026.103036 (PMC13425855; doi:10.1016/j.jacadv.2026.103036)
Supplement: Supplemental_Material [file mmc1.pdf]

Supplemental Figure 1

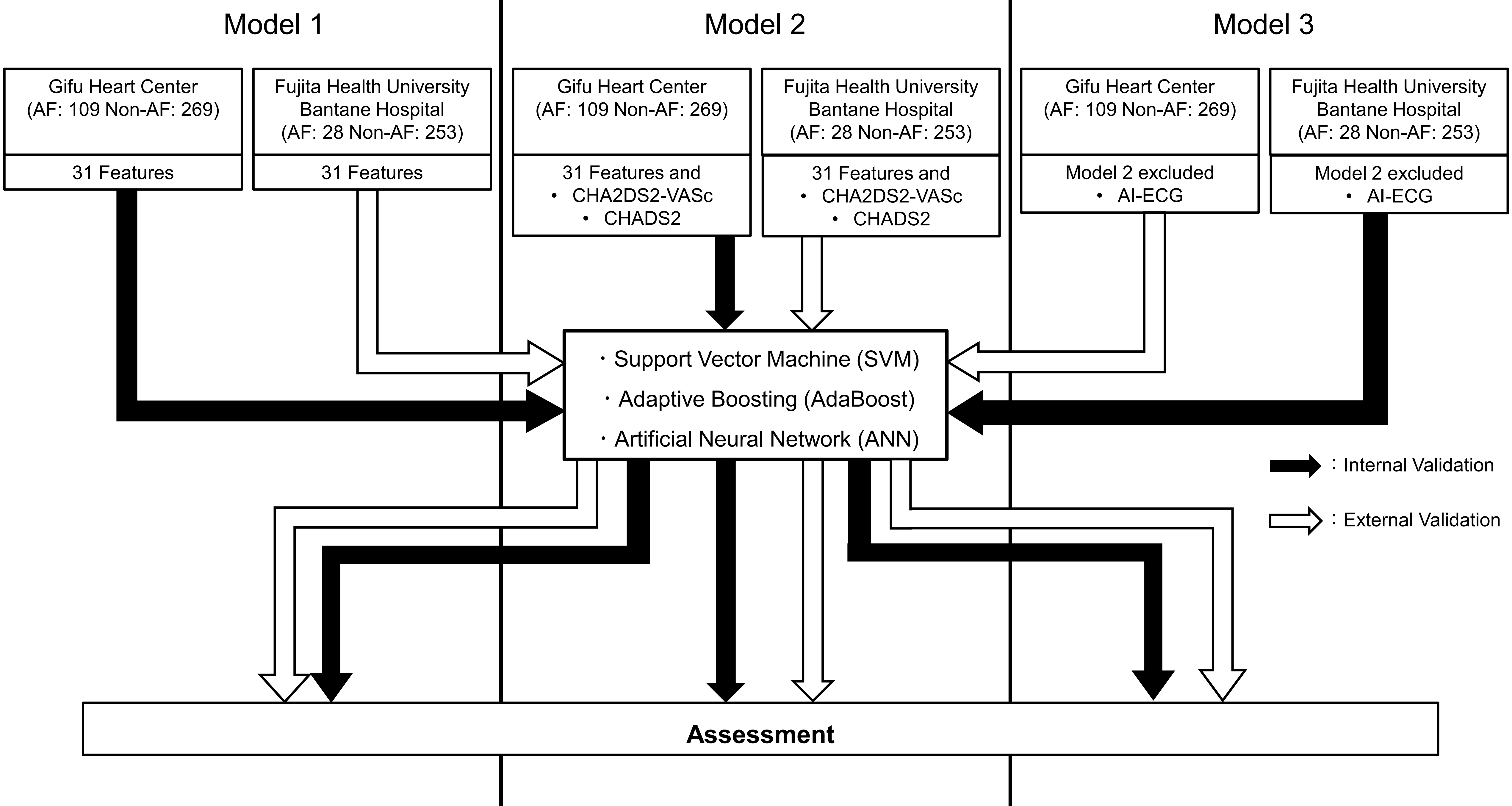

Supplemental Figure 2

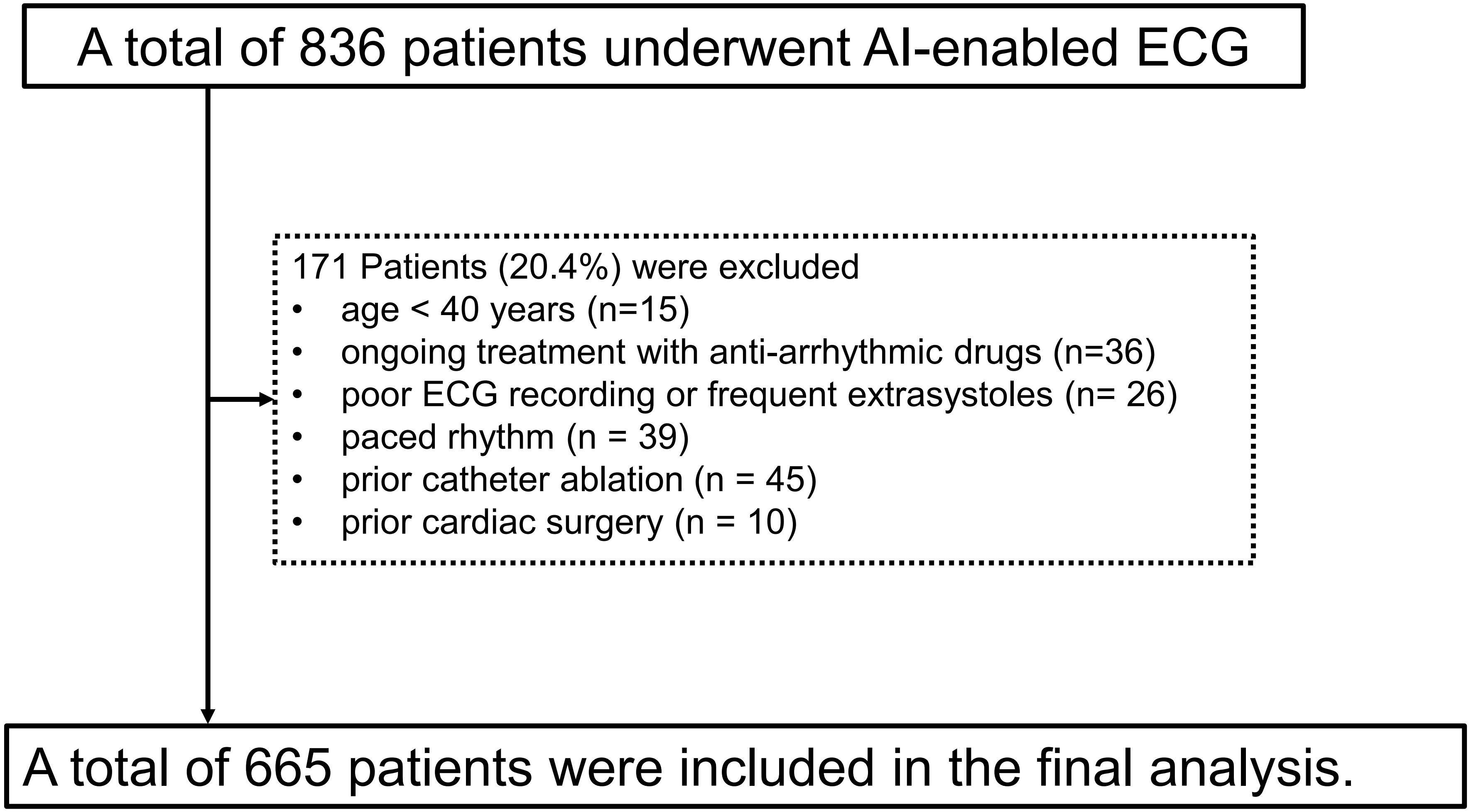

Supplemental Figure 3

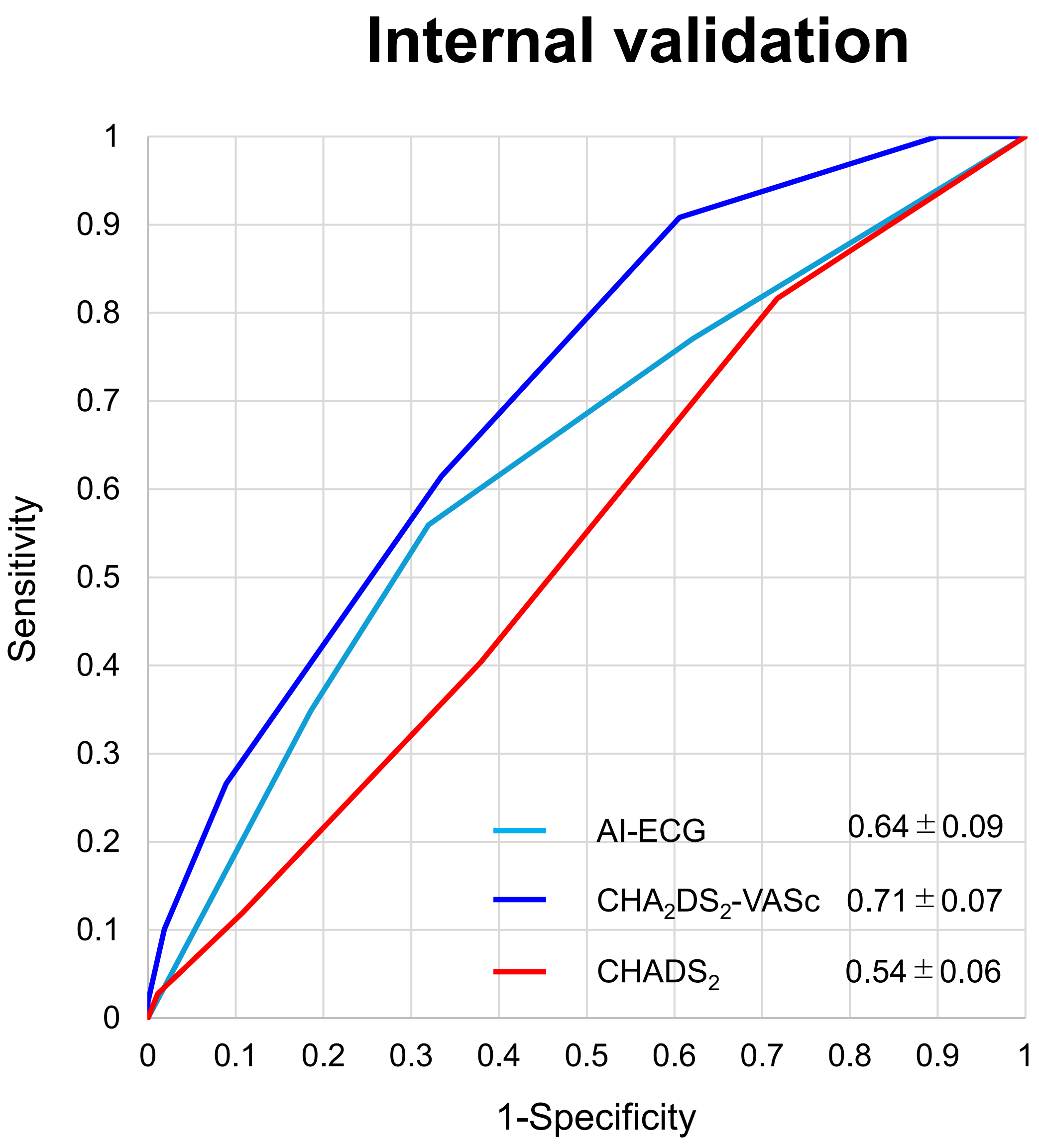

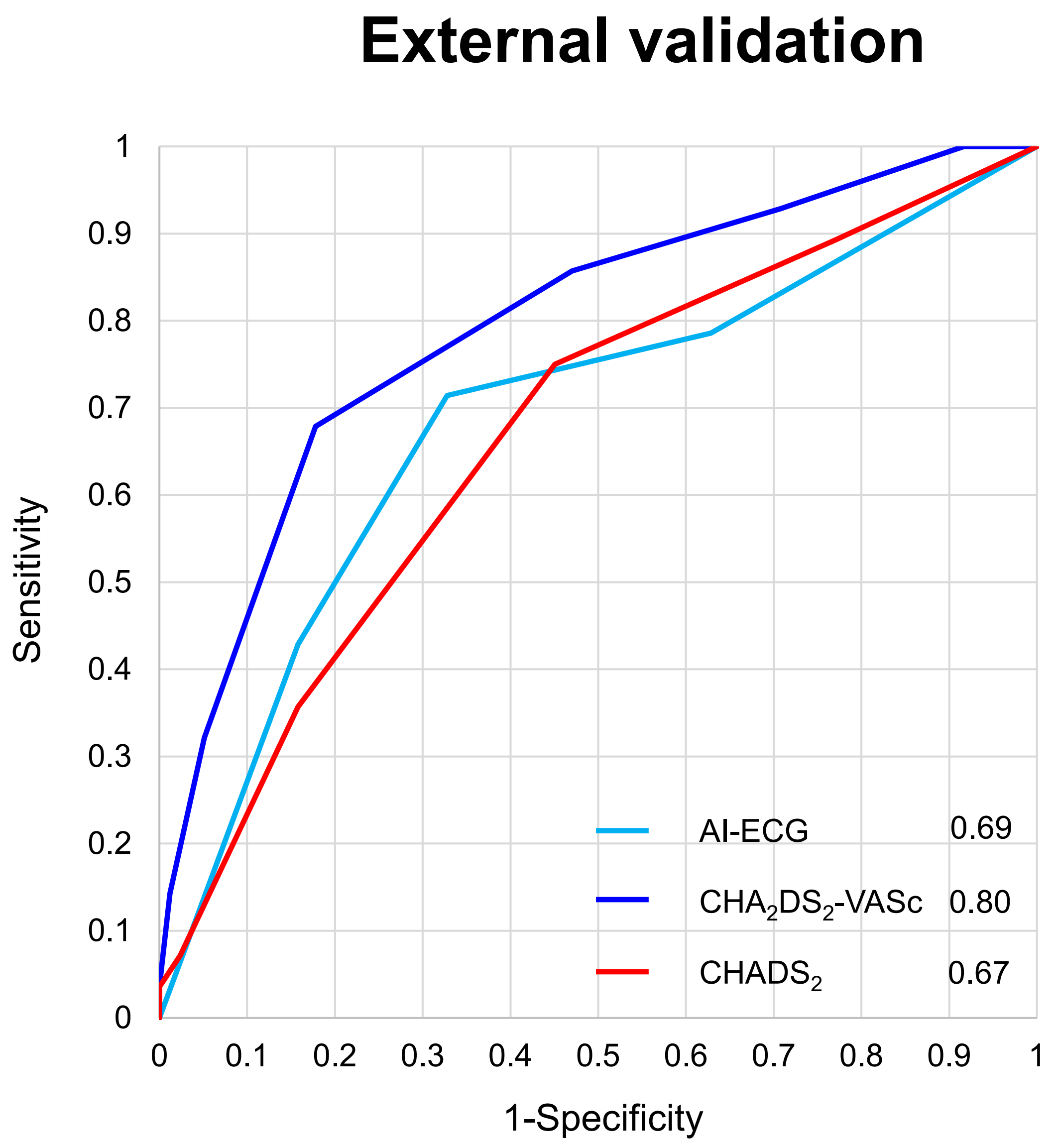

Supplemental Table 1. Patient Characteristics according to AI risk classification

	Low (n=229)	Mid-Low (n=184)	Mid-High (n=111)	High (n=141)	P-value
Age (years)	66 ± 13	68 ± 12	70 ± 12	71 ± 11	<0.01
≥65 years – no. (%)	96 (42)	65 (35)	34 (31)	34 (24)	0.01
≥75 years – no. (%)	67 (29)	69 (38)	48 (43)	66 (47)	<0.01
Female sex – no. (%)	98 (43)	70 (38)	54 (49)	63 (45)	0.32
BMI	23 ± 4	23 ± 4	24 ± 4	24 ± 4	0.55
Comorbidities – no. (%)					
CHF	13 (6)	25 (14)	18 (16)	18 (13)	<0.01
Hypertension	121 (53)	95 (52)	67 (61)	91 (65)	0.06
Diabetes Mellitus	45 (20)	44 (24)	24 (22)	32 (23)	0.75
Stroke or TIA	12 (5)	17 (5)	6 (5)	12 (9)	0.33
Vascular disease	86 (38)	65 (36)	43 (39)	42 (30)	0.39
$CHADS_2$	1.2 ± 1.1	1.4 ± 1.1	1.5 ± 1.0	1.6 ± 1.2	<0.01
CHA_2DS_2-VASc	2.1 ± 1.3	2.3 ± 1.4	2.7 ± 1.4	2.7 ± 1.5	<0.01
SBP (mmHg)	133 ± 19	135 ± 22	136 ± 22	131 ± 18	0.26
DBP (mmHg)	76 ± 14	75 ± 12	75 ± 13	72 ± 12	0.02
Heart rate – beats/min	71 ± 10	68 ± 11	67 ± 13	64 ± 12	<0.01
Laboratory data					
RBC – 10^4/μl	438 ± 60	438 ± 69	439 ± 51	436 ± 57	0.98
Hemoglobin – g/dl	13.5 ± 2.4	13.5 ± 1.6	13.5 ± 1.5	13.2 ± 1.8	0.33
BUN – mg/dl	16.8 ± 4.3	17.5 ± 8.1	16.9 ± 5.3	18.1 ± 7.5	0.60
Serum creatinine – mg/dl	0.89 ± 0.73	0.95 ± 0.87	0.91 ± 0.69	1.01 ± 0.85	0.52
eGFR – mL/min/1.73 m^2	69 ± 20	66 ± 19	63 ± 17	61 ± 21	<0.01
NT-pro BNP – pg/ml	93 (45-179)	126 (47-371)	121 (54-297)	152 (63-428)	<0.01
Electrocardiogram					
RR (ms)	865 ± 129	901 ± 140	919 ± 167	964 ± 161	<0.01
PR (ms)	167 ± 28	173 ± 30	172 ± 25	184 ± 32	<0.01
QRS (ms)	102 ± 16	104 ± 18	101 ± 15	103 ± 18	0.41
QT (ms)	389 ± 28	402 ± 34	406 ± 36	416 ± 35	<0.01
QTc	420 ± 21	425 ± 28	426 ± 28	425 ± 28	0.07
Echocardiography					
Ejection fraction (%)	60 ± 11	58 ± 13	60 ± 9	62 ± 9	0.01

Left atrial diameter (mm)	35 ± 5	37 ± 6	37 ± 7	38 ± 7	<0.01
LAVi (ml/m^2)	29 ± 10	32 ± 14	31 ± 11	37 ± 12	<0.01
IVS (mm)	9 ± 2	9 ± 2	9 ± 2	10 ± 2	0.34
PW (mm)	9 ± 1	9 ± 2	9 ± 1	9 ± 2	0.36

Abbreviations are as in Table 1.

Supplemental Table 2. Variance inflation factor analysis for the 33-feature model.

Variable	VIF
Age	3.26
Female	3.72
Height	23.75
Body weight	85.87
BMI	48.67
Comorbidities	
Heart failure	2.82
Hypertension	4.31
Diabetes mellitus	3.19
Stroke/TIA	1.50
Vascular disease	2.86
$CHADS_2$	15.46
CHA_2DS_2-VASc	9.91
Systolic blood pressure	1.78
Diastolic blood pressure	1.80
Heart rate	56.14
Laboratory data	
RBC	1.77
Hb	1.75
BUN	1.38
Cr	2.61
eGFR	2.92
NT-pro BNP	1.79
Electrocardiogram	
RR	152.14
PR	1.41
QRS	1.53
QT	359.46
QTc	165.52
Echocardiography	
EF	1.39
Left atrial diameter	2.63
LAVi	2.52

IVS	1.87
PW	1.72
AI-ECG	1.39

Abbreviations are as in Table 1.

Supplemental Table 3. SHAP Feature Importance Without $CHADS_2$ and CHA_2DS_2-VASc.

Feature	Mean (|SHAP value|)		
	SVM	AdaBoost	ANN
Age	0.26	0.46	0.02
Female	0.58	0.04	0.04
Height	1.76	0.12	0.09
Body weight	0.12	0.63	0.02
BMI	0.10	0.40	0.03
Comorbidities			
Heart failure	0.26	0.04	0.01
Hypertension	0.36	0.07	0.03
Diabetes mellitus	0.03	0.02	0.02
Stroke/TIA	0.44	0.02	0.01
Vascular disease	0.95	0.83	0.11
Systolic blood pressure	0.04	0.43	0.01
Diastolic blood pressure	0.46	0.37	0.02
Heart rate	0.37	0.14	0.02
Laboratory data			
RBC	0.64	0.35	0.04
Hb	1.07	0.51	0.07
BUN	0.30	0.23	0.02
Cr	0.61	0.16	0.04
eGFR	0.34	0.23	0.02
NT-pro BNP	0.02	0.80	0.01
Electrocardiogram			
RR	0.01	0.50	0.02
PR	0.26	0.44	0.03
QRS	1.01	0.50	0.06
QT	1.02	0.44	0.03
QTc	1.62	1.08	0.11
Echocardiography			
EF	0.02	0.18	0.01
Left atrial diameter	0.88	0.67	0.02
LAVi	0.52	0.41	0.03

IVS	0.22	0.37	0.03
PW	0.00	0.20	0.01
AI-ECG	0.66	0.59	0.06

Abbreviations are as in Table 1.

Supplemental Table 4. SHAP Feature Importance Including $CHADS_2$ and CHA_2DS_2-VASc

Feature	Mean (|SHAP value|)		
	SVM	AdaBoost	ANN
Age	0.38	0.53	0.04
Female	3.67	4.43	0.11
Height	0.93	0.83	0.11
Body weight	0.16	0.21	0.01
BMI	0.12	0.13	0.01
Comorbidities			
Heart failure	0.35	0.08	0.03
Hypertension	0.57	0.07	0.05
Diabetes mellitus	0.43	0.09	0.03
Stroke/TIA	0.14	0.07	0.03
Vascular disease	3.81	5.72	0.21
$CHADS_2$	5.57	5.80	0.17
CHA_2DS_2-VASc	9.02	9.86	0.31
Systolic blood pressure	0.01	0.29	0.02
Diastolic blood pressure	0.13	0.22	0.02
Heart rate	0.12	0.13	0.02
Laboratory data			
RBC	0.42	0.20	0.02
Hb	0.44	0.19	0.03
BUN	0.09	0.26	0.01
Cr	0.17	0.20	0.02
eGFR	0.05	0.20	0.02
NT-pro BNP	0.01	0.63	0.01
Electrocardiogram			
RR	0.04	0.42	0.01
PR	0.12	0.50	0.02
QRS	0.26	0.17	0.06
QT	0.26	0.11	0.02
QTc	0.31	0.37	0.02
Echocardiography			
EF	0.03	0.51	0.02

Left atrial diameter	0.42	0.21	0.02
LAVi	0.09	0.26	0.02
IVS	0.01	0.31	0.03
PW	0.02	0.24	0.02
AI-ECG	0.25	0.33	0.02

Abbreviations are as in Table 1.

Supplemental Table 5. Incremental value of AI-ECG for AF classification.

Predictive models	AUC (95% CI)	NRI	Z score	P-value
Model 1 (CHA_2DS_2-VASc)	0.69 (0.64–0.73)	Ref	—	—
Model 2 (CHA_2DS_2-VASc + AI-ECG)	0.71 (0.67–0.76)	0.007	0.10	0.92

Model 1 was constructed using CHA_2DS_2-VASc alone, whereas Model 2 incorporated both CHA_2DS_2-VASc and AI-ECG. NRI indicates net reclassification improvement; CI, confidence interval.
